# Two roads diverged in a cell: insights from differential exosome regulation in polarized cells

**DOI:** 10.3389/fcell.2024.1451988

**Published:** 2024-09-02

**Authors:** Tadayuki Komori, Mitsunori Fukuda

**Affiliations:** Laboratory of Membrane Trafficking Mechanisms, Department of Integrative Life Sciences, Graduate School of Life Sciences, Tohoku University, Sendai, Miyagi, Japan

**Keywords:** ESCRT, exosome, extracellular vesicle, heterogeneity, multivesicular body (MVB), polarized epithelial cell, Rab small GTPase, SNARE

## Abstract

Exosomes are extracellular vesicles involved in intercellular signaling, carrying various cargo from microRNAs to metabolites and proteins. They are released by practically all cells and are highly heterogenous due to their origin and content. Several groups of exosomes are known to be involved in various pathological conditions including autoimmune, neurodegenerative, and infectious diseases as well as cancer, and therefore a substantial understanding of their biogenesis and release is crucial. Polarized cells display an array of specific functions originated from differentiated membrane trafficking systems and could lead to hints in untangling the complex process of exosomes. Indeed, recent advances have successfully revealed specific regulation pathways for releasing different subsets of exosomes from different sides of polarized epithelial cells, underscoring the importance of polarized cells in the field. Here we review current evidence on exosome biogenesis and release, especially in polarized cells, highlight the challenges that need to be combatted, and discuss potential applications related to exosomes of polarized-cell origin.

## 1 Introduction

Extracellular vesicles (EVs) are defined as “particles that are released from cells, are delimited by a lipid bilayer, and cannot replicate on their own (i.e., do not contain a functional nucleus)” (Welsh et al., 2024). Several cellular pathways are responsible for the generation of EVs as their origins range from endosomes to the plasma membrane (PM). Virtually all cells produce EVs, making the population of EVs in any biological fluid highly heterogenous. EVs have been described in scores of functions, seemingly dependent on the origin and state of the donor cell, and are a hot topic regarding cell-to-cell communication (Kalluri and LeBleu, 2020).

From their origin or cellular pathway, EVs can ideally be classified into multiple categories (van Niel et al., 2018; van Niel et al., 2022). Probably the most famous of the EVs is the exosome, a vesicle originating from the inward budding of endosomes and released from the resulting multivesicular bodies (MVBs). The size of exosomes ranges from 30 or 50 nm to 150 or 200 nm in diameter, varying between reports. Other types of EVs include ectosomes (or microvesicles) that bud from the PM and are 50 to 10,000 nm in diameter, apoptotic bodies (50–5000 nm), and other larger vesicles (van Niel et al., 2018; van Niel et al., 2022). Due to the overlap in the size, one should note that no practical procedure is capable of separating each of the differentially produced EVs (Welsh et al., 2024). For example, a typical set of experiments aimed for purifying exosomes [in this case, differential ultracentrifugation (dUC)] would give EVs with diameters ranging roughly from 30 to 200 nm (Sinha et al., 2016; Klingeborn et al., 2017; Zhang et al., 2018; Colombo et al., 2021; Mathieu et al., 2021; Mizenko et al., 2021; Ferreira et al., 2022; Fordjour et al., 2022; Matsui et al., 2021; Ciftci et al., 2023) and a density of 1.14–1.20 g/mL sucrose (Chen et al., 2016), a possible mixture of exosomes, smaller ectosomes and other non-vesicular bodies. A strictly isolated preparation of exosomes that are triple positive of three tetraspanins (CD63, CD9, and CD81) exhibited densities of 1.05–1.15 g/mL sucrose (Jeppesen et al., 2019). For practical reasons, the term exosome will be used in this review to describe both the vesicles released from MVBs and the set of small EVs mainly under 200 nm obtained by dUC or other isolation methods, the former mainly in the context of biogenesis and the latter in functions.

An emerging topic in the world of exosomes comes from perspectives from polarized cells. These cell types were found to employ unique proteins in MVB trafficking not yet discovered to be involved in nonpolarized cells (Matsui et al., 2022). These proteins can possibly become a clue for untangling the regulation mechanism of a single type of cell releasing multiple subsets of exosomes, not only in polarized cells (van Niel et al., 2001; Sreekumar et al., 2010; Tauro et al., 2013; Chen et al., 2016; Klingeborn et al., 2017; Davies et al., 2020; Wang et al., 2021) but even in nonpolarized cells (Zhang et al., 2018; Nikoloff et al., 2021). In this review, we will first briefly discuss current knowledge on exosomes in general, from biogenesis to functions, then move on to the differences that emerge in polarized cells.

## 2 Overview of exosome biogenesis

### 2.1 General remarks

The first idea of exosomes as externalized vesicles from endosomal origin were proposed back in 1983 by the Johnstone and Stahl laboratories (Couch et al., 2021). They found that transferrin receptors in reticulocytes happened to be released on vesicles that originated in MVBs (Pan and Johnstone, 1983; Pan et al., 1985). Since then, numerous studies have unraveled the biological and pathological importance of exosomes, some of which we will discuss later in this review. Although the main interest of most scientists was the contents or functions of these exosomes, biogenesis of exosomes was also a major topic.

**FIGURE 1:**
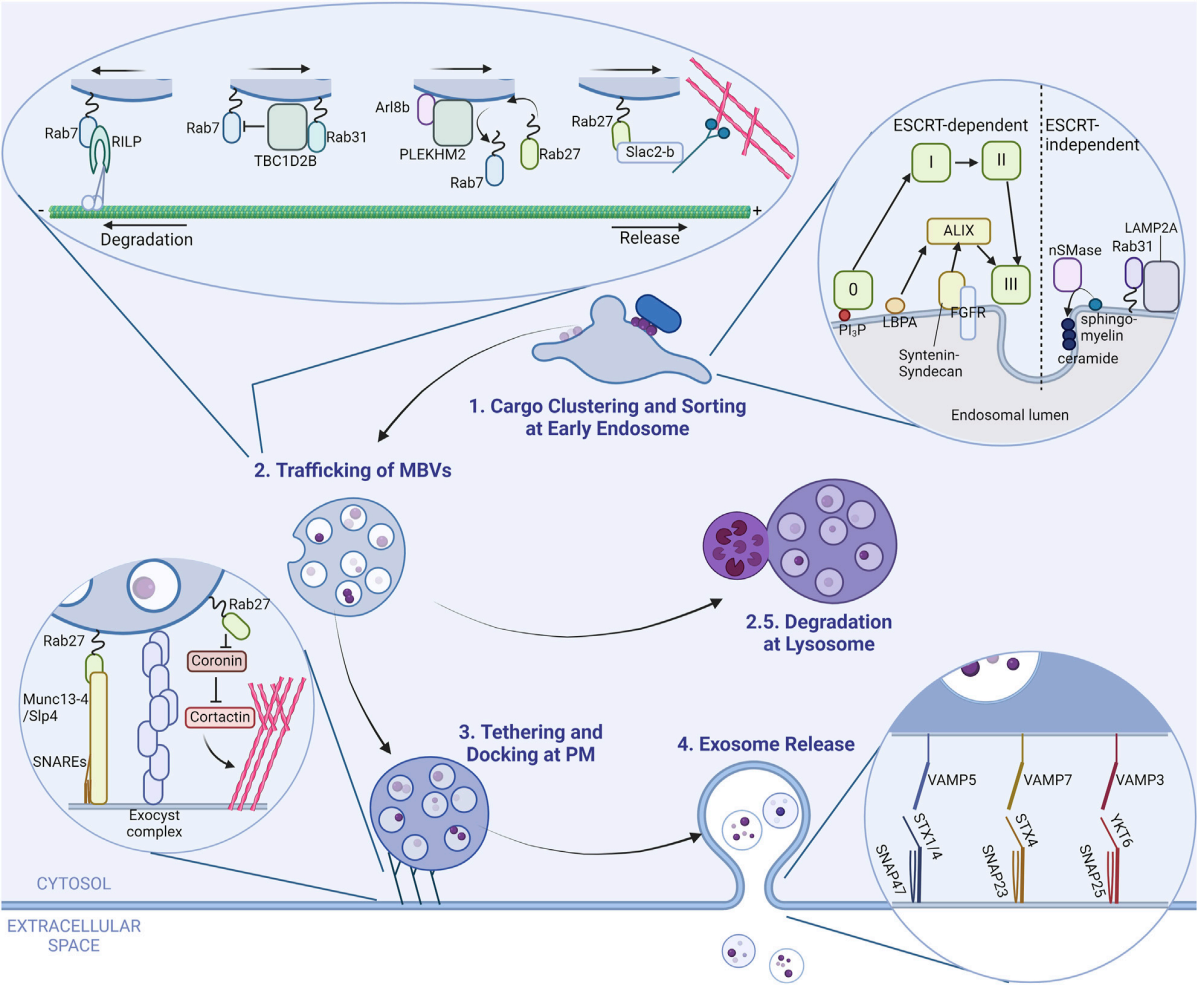
A brief overview of exosome dynamics in cell. A scheme of exosome biogenesis and release from a generic cell is illustrated in four steps. (Step 1) Exosomal cargo proteins accumulate at the early endosome membrane, which eventually bud into the lumen with the help of several different pathways and form intraluminal vesicles (ILVs). The inset shows proteins and pathways responsible for this step. The light-green squares represent ESCRT complexes. (Step 2) Multivesicular bodies (MVBs) that harbor multiple ILVs are trafficked to either the cell periphery for release or to lysosomes for degradation (Step 2.5). Proteins that regulate this step are shown in the inset. (Step 3) MVBs at the cell periphery are tethered and docked to the plasma membrane (PM) by proteins marked in the inset. (Step 4) MVBs fuse with the PM via specialized combinations of SNARE proteins to release its content. The SNAREs responsible for this step are shown in the inset.

There are roughly four steps until a typical exosome is born and released from a cell: cargo accumulation and formation of intraluminal vesicles (ILVs), sorting and maturation of MVBs, their docking to the PM, and release of exosomes (Figure 1). The fine details of exosome biogenesis are excellently reviewed in previous reviews (Juan and Fürthauer, 2018; van Niel et al., 2018; Han et al., 2022).

### 2.2 Cargo accumulation at the endosomal surface and formation of ILVs

The initial step of exosome biogenesis happens at the early endosome. Here, multiple pathways regulate cargo accumulation, depending on the cargo or cell type (Juan and Fürthauer, 2018; van Niel et al., 2018; Gruenberg, 2020; Kalluri and LeBleu, 2020; Han et al., 2022). These pathways are classified by the involvement of endosomal sorting complexes required for transport (ESCRT) complexes; hence their names ESCRT-dependent and -independent pathways. ESCRT complexes are comprised of 5 subcomplexes (ESCRT-0, -I, -II, -III and VPS4) and a total of around 30 ESCRT components that participate from cargo clustering up to ILV budding and scission. In the ESCRT-dependent pathway, ESCRT-0 is the main complex in mooring cargo to the endosomal membrane. ESCRT-0 binds to phosphatidylinositol 3-phosphate (PI3P), a lipid enriched on endosomal membranes, on one side and ubiquitinated cargo on the other (Gruenberg, 2020; Han et al., 2022). This then recruits ESCRT-I and -II, which are scaffolds for ESCRT-III polymerization (Schöneberg et al., 2017; Juan and Fürthauer, 2018; Gruenberg, 2020; Han et al., 2022). Polymerized ESCRT-III has the capability to deform and cut membranes, resulting in the formation of ILVs (Schöneberg et al., 2017).

Other pathways that are partially dependent on ESCRT complexes exist. The key molecule in those pathways is ALG-2-interacting protein X (ALIX) (Han et al., 2022), hence the name ALIX-dependent pathway or non-canonical ESCRT-dependent pathway. The ALIX-dependent pathway recognizes different cargoes than the ESCRT-dependent pathway and start with different proteins on the endosomal surface such as syndecan–syntenin which recognize FGFR and binds to ALIX (Baietti et al., 2012; Han et al., 2022) or lysobisphosphatidic acid (LBPA) together with tetraspanins such as CD63 and CD9 that also bind to ALIX, although the exact cargo remains to be determined (Larios et al., 2020; Han et al., 2022). Either way, ALIX binds to cargo receptors and recruits ESCRT-III to facilitate ILV formation (van Niel et al., 2018; Gruenberg, 2020; Han et al., 2022). All the mentioned pathways are depicted in the inset of Figure 1; however, it is important to note that there are additional pathways involved in MVB formation.

There are several pathways for cargo accumulation that are in turn ESCRT-independent. The key components of ESCRT-independent pathways are ceramides (Han et al., 2022), which are a type of lipid found on organelle membranes and participate in various cell signaling pathways. In the context of ILV formation, ceramides generated from other sphingolipids gather around themselves and create a negative curvature, inducing membrane budding, at least *in vitro* (Trajkovic et al., 2008). Although there might be other components regulating this spontaneous formation of ILVs that are not found yet, most proteins involved in ESCRT-independent pathways interact with ceramides or ceramide-interacting proteins. One of the pathways involve neutral sphingomyelinase 2 (nSMase2) which converts sphingomyelin to ceramides. This pathway is reported to be responsible for packaging some sorts of prions and aggregates (Guo et al., 2015; Dinkins et al., 2016; Miranda et al., 2018) or microRNAs (miRNAs) (Kosaka et al., 2013; Barman et al., 2022). One unusual cargo dependent of nSMase2 is a V-ATPase subunit ATP6V0A1. This subunit is responsible for acidification of MVBs, and incorporation of this subunit in ILVs promotes secretion of exosomes, but by an unknown mechanism (Choezom and Gross, 2022). Another pathway involves Rab31 (also known as Rab22B), where flotillin-1 and LAMP2A sequester ubiquitinated receptor tyrosine kinases and tetraspanins or proteins with a signal (KFERQ or similar) sequence, respectively, and target them for exosomal release (Wei et al., 2021; Ferreira et al., 2022; Han et al., 2022).

### 2.3 Trafficking of MVBs

MVBs loaded with ILVs are then sorted and transported either to the PM for exosome release or to lysosomes for degradation. MVBs are late endosomes and thus decorated with Rab7 (Vanlandingham and Ceresa, 2009). Without any interventions, MVBs are directed towards a degradative fate (to the minus end of microtubules, which is toward lysosomes) by the Rab7 effector Rab-interacting lysosomal protein (RILP) (van Niel et al., 2018; Han et al., 2022). As depicted in the inset in Figure 1, some proteins are responsible for evading this fate like Rab31 or Arl8b. Rab31 recruits TBC1D2B, a Rab7 GTPase-activating protein (GAP), deactivating Rab7 and directing MVBs away from degradation (Wei et al., 2021). Arl8b is another small GTPase known to remove Rab7 from membranes by recruiting SifA and kinesin-interacting protein (SKIP, also called PLEKHM2) (Jongsma et al., 2020). Arl8b on MVBs acts in the same manner, removing Rab7 from the surface and making room for Rab27A and Rab27B to associate to MVBs, directing MVBs to the cell periphery away from lysosomes (Verweij et al., 2022). Rab27A and Rab27B are one of the main Rabs that traffic MVBs to the cell periphery (the plus ends of microtubules) along with their effector Slac2-b (Ostrowski et al., 2010), mainly via increased association with actin filaments (Ostrowski et al., 2010).

Other proteins are also reported to be involved in trafficking of MVBs, such as Rab11 (Savina et al., 2005; Bai et al., 2022; Marie et al., 2023; Wells et al., 2023) and Rab35 (Hsu et al., 2010; Zhang et al., 2022). Rab11 is implicated in some types of cells as a necessary component in homotypic fusion of MVBs (Savina et al., 2005) and trafficking to the PM (Bai et al., 2022), in addition to its role in a relatively slow recycling of membranous proteins to the PM. Regulation of MVBs by Rab11 in specific cells might be due to the difference in the origin of MVBs, as MVBs were found to also originate from recycling endosomes positive of Rab11 in some types of cancer cells or *Drosophila* cells (Fan et al., 2020; Marie et al., 2023; Wells et al., 2023). Rab35 regulates exosome release along with its GAP, TBC1D10A–C, but with rather unclear mechanisms (Hsu et al., 2010; Zhang et al., 2022).

### 2.4 Tethering and docking of MVBs to the plasma membrane

Mature MVBs that evaded the fate of degradation are tethered and docked to the PM by the exocyst complex (Bai et al., 2022) or by Slp4 and Munc13-4, effectors of Rab27 (Fukuda, 2006; Ostrowski et al., 2010; Boswell et al., 2012; He et al., 2016). Both Slp4 and Munc13-4 bind directly to the PM through their phospholipid binding activity and also indirectly bind to the PM through interaction with soluble *N*-ethylmaleimide-sensitive factor attachment protein receptor (SNARE) proteins. Interactions with branched actin filaments at the cell periphery are also suggested as a mechanism for docking of MVBs to the PM (Hoshino et al., 2013). Rab27A is known to also play a positive role in regulation of actin branching as well by inhibiting coronin, which is an inhibitor of cortactin, the main protein in actin branching (Sinha et al., 2016). These tethers are illustrated in the inset of Figure 1.

### 2.5 Release of exosomes

After tethering and docking to the PM, SNARE proteins specific for MVB–PM fusion, depending on the cells, for example, the set of vesicle-associated membrane protein 5 (VAMP5), synaptosome-associated protein 47 (SNAP47) and syntaxin-1 (STX1) or STX4 (Matsui et al., 2023) or VAMP7, SNAP23, and STX4 (Williams et al., 2014; Liu et al., 2023), as well as other sets including VAMP3, YKT6 or SNAP25 (van Niel et al., 2018; Han et al., 2022) fuse the MVB with the PM, releasing the content which are now called exosomes. The reported combinations of SNARE proteins are summarized in the inset of Figure 1.

## 3 Exosome components and functions

As one can see from the many pathways of exosome biogenesis, exosome components include highly regulated cargo as well as specific regulatory or membranous components. Extensive research on the composition of exosomes and related EVs have revealed specific proteins and cargoes for them (Zhang et al., 2018; Jeppesen et al., 2019; Lischnig et al., 2022; Seo et al., 2022). Typical exosomes are positive of three tetraspanins (CD63, CD9, and CD81) (Kowal et al., 2016; van Niel et al., 2018; Zhang et al., 2018; Jeppesen et al., 2019; Lischnig et al., 2022; Seo et al., 2022), proteins involved in their biogenesis (ALIX, TSG101, syntenin-1) (Kowal et al., 2016; Zhang et al., 2018; Jeppesen et al., 2019; Lischnig et al., 2022; Seo et al., 2022), functional proteins [e.g., transcription factors, cytokines, or heat shock proteins (van Niel et al., 2018; Kalluri and LeBleu, 2020; Ferreira et al., 2022)], and although controversial (Chevillet et al., 2014; Jeppesen et al., 2019), a variety of miRNAs (van Niel et al., 2018; Kalluri and LeBleu, 2020; Fiorani et al., 2023). A comprehensive collection of proteomic (and other molecular) data from studies involving exosomes and other EVs could be found in the vesiclepedia website (Kalra et al., 2012; Pathan et al., 2019; Chitti et al., 2023). On the other hand, exosomes were found to be void of typical cytosolic proteins such as cytoskeletal proteins, glycolytic and metabolic enzymes, or chaperones (Jeppesen et al., 2019), indicating a strictly regulated cargo loading mechanism.

**FIGURE 2:**
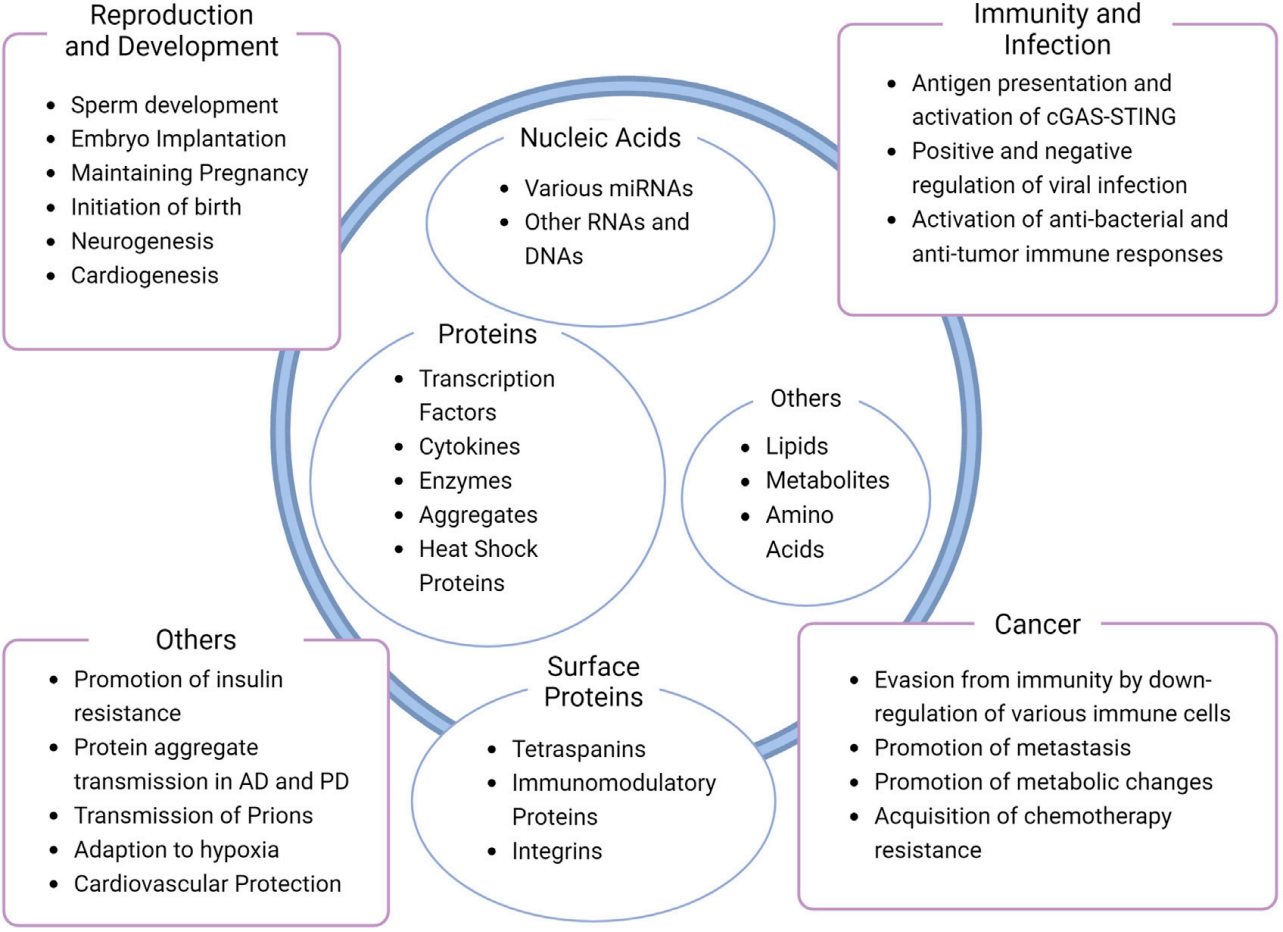
Diversity of exosome composition and function. Common or well-known contents of exosomes and functions are shown inside and outside the schematic exosome, respectively (Kowal et al., 2016; van Niel et al., 2018; Zhang et al., 2018; Jeppesen et al., 2019; Hashimoto et al., 2020; Kalluri and LeBleu, 2020; Ferreira et al., 2022; Lischnig et al., 2022; Seo et al., 2022; Fiorani et al., 2023; Korvenlaita et al., 2023; Ma et al., 2024). Note that the figure is not a comprehensive collection of exosome contents or functions, nor a representative of a single exosome.

Exosomes exhibit a wide array of functions that originate from their diverse content. Physiological aspects of exosomes can be observed in reproduction or development, whereas the pathological counterpart is related to phenomena from infection and immunity to progression in cancer and neurodegenerative, metabolic, and cardiovascular diseases (nicely reviewed in (Kalluri and LeBleu, 2020), additionally, (Hashimoto et al., 2020; Ferreira et al., 2022; Fiorani et al., 2023; Korvenlaita et al., 2023; Ma et al., 2024). In the context of inflammation, antigen presenting cells may release exosomes containing major histocompatibility complex (MHC) carrying antigenic peptides. These exosomes can activate specific T cells for immune responses (Raposo et al., 1996; Kalluri and LeBleu, 2020). Another example of exosomal function would be their role in pregnancy. At different stages of pregnancy, exosomes containing different cargo are secreted from various cells to either initiate embryo implantation, maintain pregnancy or trigger delivery (Hashimoto et al., 2020). A few of the functions of exosomes are organized and listed in Figure 2.

Another interesting feature of exosomes is their organotropism. Exosomes from cancer cells have specific integrin protein sets that facilitate their association and uptake to tissues and cells that harbor specific extracellular matrix proteins (Hoshino et al., 2015). Whether this phenomenon is specific to certain types of exosomes remain unclear, but exosome uptake by specific cell types should also be considered an important characteristic of exosomes.

## 4 The “two roads” diverged in the cell: differential regulation of exosomes in polarized cells

### 4.1 General remarks

**FIGURE 3:**
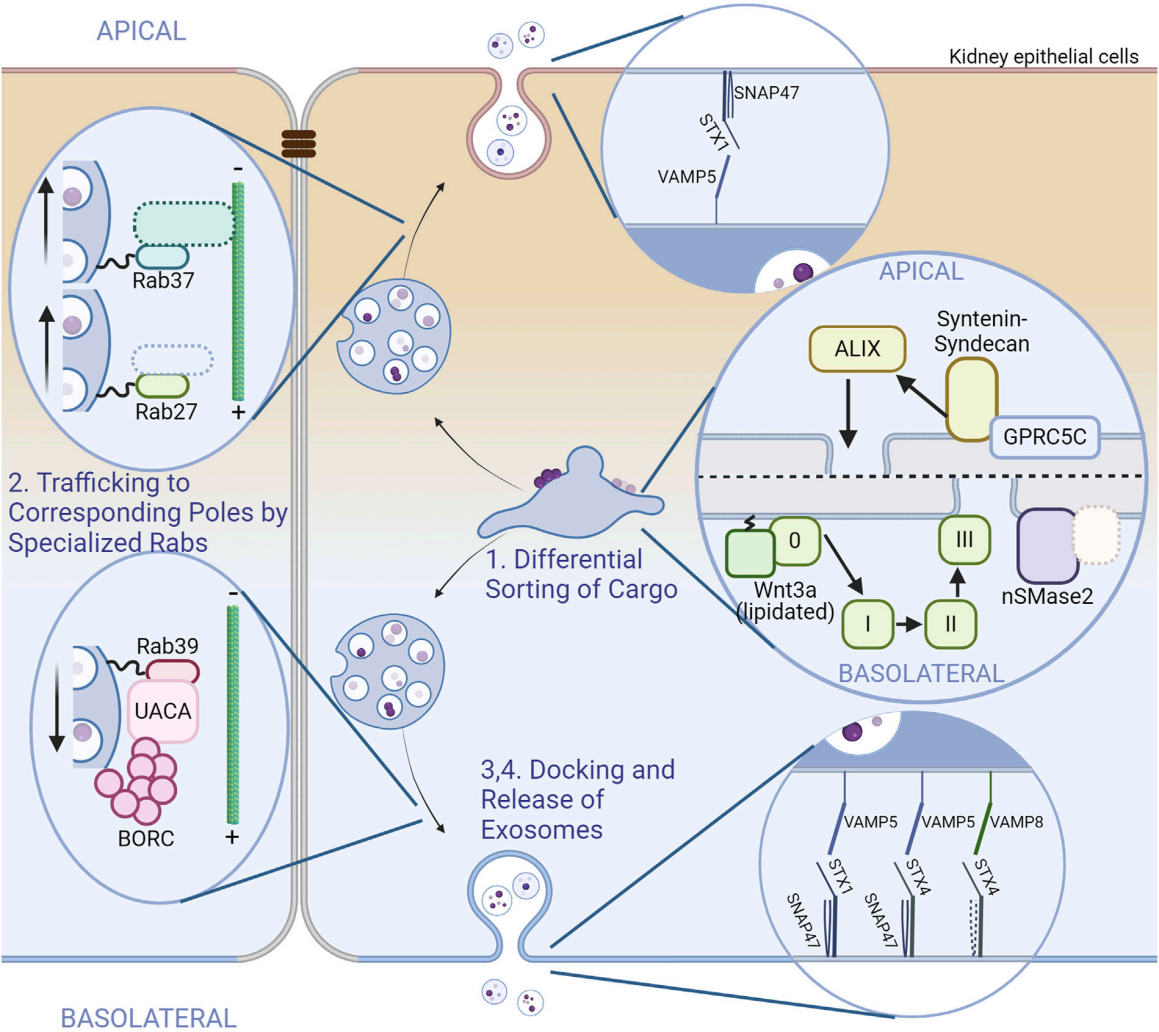
Exosome dynamics and comparison of proteins involved between poles in polarized cells. A scheme of exosome biogenesis and release from renal (kidney) epithelial cells is illustrated. The apical side is on the upper side and the basolateral side is on the lower side. Tight junctions are depicted between cells for clarity. Unidentified or hypothetical proteins are marked with dotted boundaries. The steps of exosome biogenesis are shown as in Figure 1, along with the insets. Some steps were omitted due to the lack of reports. (Step 1) Sorting and accumulation of exosomal cargo depends on different pathways. The apical side utilizes the ALIX–syntenin–syndecan pathway without clear involvement of the conventional ESCRT complexes. The basolateral side uses the ESCRT-dependent pathway for cargoes such as lipidated Wnt3, and nSMase2 with unidentified cargoes (Step2) MVBs intended for exosomal release are trafficked to the corresponding poles by corresponding Rab proteins. Rab27 and Rab37 traffic MVBs to the apical side. Effectors are not determined for Rab37; Involvement of known Rab27 effectors have not been investigated in polarized cells. For the basolateral side, Rab39 and its effector UACA traffics MVBs with the help of BORC. (Step 4) The release of exosomes also depend on specific combinations of SNARE proteins. The combinations are shown in the insets (Chen et al., 2016; Colombo et al., 2021; Matsui et al., 2021; Matsui et al., 2022; Matsui et al., 2023).

Given that exosomes are highly heterogenous and that practically all types of cells release exosomes, one could infer that there exists some distinction in the subtypes of exosomes released from a single type of cell, and that they are regulated by different pathways. The first report that shows asymmetrical exosome release from polarized cells dates back to 2001, but it was not until recently that various studies started to emerge showing there were at least two subtypes of exosomes released from each of the apical and basolateral sides of polarized epithelial cells. These subpopulations of exosomes were found to have different compositions (van Niel et al., 2001; Sreekumar et al., 2010; Tauro et al., 2013; Chen et al., 2016; Klingeborn et al., 2017; Davies et al., 2020; Wang et al., 2021). Research has also dug into the mechanisms why there might be these differences (Colombo et al., 2021; Matsui et al., 2021). Further studies highlighted the differences in intracellular trafficking of the MVBs where different Rab proteins were in charge of the apical and basolateral MVBs (Matsui et al., 2022). From these evidence, we can see that the different subpopulations of exosomes a single cell can produce are regulated differently from a very early point of biogenesis. However, there are still several unknown and unresolved pathways and processes an exosome goes through before being released by a polarized cell, whether it be from the apical or basolateral side. The next few paragraphs will be on the exosome biogenesis pathway, giving a better view of those specific to polarized cells by comparing them with conventional pathways. The differential proteins in each step are shown in Figure 3.

### 4.2 Cargo accumulation at the endosomal surface and formation of ILVs

The earliest point of exosome biogenesis, the accumulation of cargo at early endosomes, is not in its fully clarified state, but there are some understandings based on the various pathways a polarized cell uses for cargo accumulation. For instance, a study on Wnt proteins released in apical or basolateral exosomes from polarized Mardin-Darby canine kidney (MDCK) cells has provided us with evidence that basolateral exosomes are likely formed by the ESCRT-dependent pathway, as these exosomes were specifically associated with tumor susceptibility gene 101 (TSG101), a component of the ESCRT-I subcomplex. As basolateral exosomes carry lipidated Wnt3a whereas apical exosomes carry nonlipidated Wnt3a (Chen et al., 2016), the ESCRT-dependent pathway might be responsible specifically for the cargo loading of basolateral exosomes in these cell types. Another study has revealed that TSG101 was enriched more in apical exosomes than basolateral exosomes in retinal pigmented epithelial (RPE) cells (Klingeborn et al., 2017). A cargo that closely correlated with TSG101 was SLC39A12, which is likely to be regulated by the ESCRT-dependent pathway. Notably, in both studies, the exosome that correlated with ESCRT components had a density comparable to that of conventional exosomes (the apical exosome of MDCK cells had higher densities and the basolateral exosomes of RPE cells had lower densities). A further study on ILV biogenesis in MDCK cells have revealed more on the pathways each exosome uses. Knockdowns of several components of ILV-forming pathways have successfully pinpointed at ALIX–syntenin1–syndecan1 and nSMase2 as responsible components for apical and basolateral exosome biogenesis, respectively (Matsui et al., 2021). A cargo likely to be regulated by the ALIX–syntenin1–syndecan1 pathway is GPRC5C, a G protein-coupled receptor that sense the lack of natural sugars (Kawabata et al., 2023), only found in the apical exosome (Matsui et al., 2021). These studies indicate that populations of exosomes released pole-specifically in certain cell types are generated through different mechanisms. The involvement of other molecules such as LBPA and tetraspanins in the ALIX-dependent pathway or Rab31 and LAMP2A in the ESCRT-independent pathway has not been elucidated yet. There also might be novel pathways undiscovered in polarized cells that also function in conventional cell types.

### 4.3 Trafficking of MVBs

Trafficking of the MVBs to their corresponding poles involves several Rab proteins and their effectors not yet documented in conventional exosome biogenesis. The main Rab in this step in nonpolarized cells was Rab11, Rab27, and Rab35 as mentioned above. In polarized cells, or at least in epithelial cells, Rab27 is only involved in the trafficking of MVBs to the apical side along with another Rab, Rab37, which is phylogenetically similar to Rab27 (Homma et al., 2021). Knockdowns of these Rabs had an additive effect, suggestive of a redundant or parallel trafficking of MVBs (Matsui et al., 2022). As the microtubule organizing center (MTOC) of epithelial cells is at the apical surface, the direction of trafficking MVBs are inverted from generic cells in apical exosome release; The MVBs at the apical side move from the plus end to the minus end of microtubules for exosome release. Whether Rab27 and Rab37 regulate different populations of exosomes or how Rab37 controls MVB trafficking are still a mystery.

In epithelial cells, MVB trafficking to the basolateral side is governed by Rab39. Rab39 interacts with uveal autoantigen with coiled-coil domains and ankyrin repeats (UACA), which binds to BLOC-1-related complex (BORC) (Matsui et al., 2022). BORC is a complex found to traffic lysosomes to the cell periphery (Pu et al., 2015). Epithelial cells may also utilize this machinery for MVBs, as knockdowns of BORC components resulted in a decrease in exosome release from both sides (Matsui et al., 2022). The involvement of Rab31 or Arl8b in MVB trafficking in polarized cells are not yet assessed in neither of the poles.

### 4.4 Tethering and docking of MVBs to the plasma membrane

No specific tether (or tethering factor) has been described for exosome release from either of the poles. Although undescribed, the Rab27 effectors, Slp4 and Munc13-4, might function as tethers only at the apical side given that Rab27 is not involved in basolateral exosome trafficking and release (Matsui et al., 2022). A similar mechanism might exist at the basolateral surface with Rab39 effectors.

### 4.5 Release of exosomes

Regarding the SNAREs, one SNARE is already found to be specialized for polarized exosome release. VAMP8 is a v-SNARE that regulates basolateral exosome release in MDCK cells (Colombo et al., 2021). One of the target SNAREs of VAMP8, STX4, resides selectively on the basolateral membrane (Gaisano et al., 1996; Low et al., 1996; Fujita et al., 1998). As stated earlier, STX4 is a SNARE responsible for exosome release in conventional cells, and it was also found later that STX4 indeed specifically controlled basolateral exosome release in MDCK cells, although with the set of VAMP5 and SNAP47 (Matsui et al., 2023). This set of VAMP5 and SNAP47 was also found to be responsible for regulating the release of exosomes from both sides with the counterpart STX1 (Matsui et al., 2023). The v-SNARE specifically responsible for apical exosome release has not been clarified yet. A t-SNARE localized at the apical membrane, STX3, which is closely related to STX4 (Gaisano et al., 1996; Low et al., 1996; Fujita et al., 1998; ter Beest et al., 2005), could be a factor in apical exosome release, although its participation in exosomal release in general has not been described.

In other polarized cells, for example, immune cells with immunological synapses or neuronal cells, there are only indirect evidence of polarized exosome release, mainly from the intracellular localization changes of MVBs (Mittelbrunn et al., 2015) or exosome isolation upon immune activation (Herranz et al., 2019; Bello-Gamboa et al., 2020; Calvo and Izquierdo, 2020). Although there are great limitations to differential isolation of polarized exosomes in these types of cells, further investigation could also give us more hints on the differential regulation of releasing different subsets of exosomes.

## 5 Exosomes from polarized cells and their reported functions

As mentioned above, different subtypes of exosomes released from different sides of polarized cells have different protein compositions (van Niel et al., 2001; Sreekumar et al., 2010; Tauro et al., 2013; Chen et al., 2016; Klingeborn et al., 2017; Davies et al., 2020; Colombo et al., 2021; Matsui et al., 2021; 2022; Wang et al., 2021). We are on the verge of starting to understand their differential functions. Here we will summarize current knowledge on some of the reported functions specific of apical or basolateral exosomes and the molecules behind them.

One of the first reports of exosomes from polarized cells focused on the immunological aspects of exosomes (van Niel et al., 2001). Both apical and basolateral exosomes released from human intestine-derived cell lines carried MHC class I and II, but with a higher abundance in basolateral exosomes. Additionally, a mass spectrometry of sample-specific bands of exosomal lysates (note: not exosomal proteome analysis) revealed protein profiles of apical and basolateral exosomes in these cell lines, although unfortunately no further data on the functions of these exosomes were given.

Other cells that have gone under close examination of their spectra of exosomes are colon carcinoma organoids (Tauro et al., 2013), RPE cells (Sreekumar et al., 2010; Klingeborn et al., 2017), MDCK cells (Chen et al., 2016; Colombo et al., 2021; Matsui et al., 2021; 2022), human primary proximal tubular epithelial cells (PTEC) (Wang et al., 2021), and human cholangiocytes (Davies et al., 2020). The differences in the proteomic (Tauro et al., 2013; Klingeborn et al., 2017; Wang et al., 2021) and miRNA (Davies et al., 2020; Wang et al., 2021) contents or post-translational modifications (PTMs) (Chen et al., 2016) between the subtypes have also been recorded, but the characteristics of these exosomes seem to vary greatly from cell to cell. For example, the proteins enriched in apical exosomes from RPE cells and colon carcinoma cells had very little overlap except the tetraspanins on the exosomal surface (Tauro et al., 2013; Klingeborn et al., 2017), although the methods used to analyze relevant proteins in exosomal fractions might be the cause. Due to this diversity, no generalized view of the characteristics of apical or basolateral exosomes can currently be built. One explanation of this diversity might be because the mechanism in the acquisition of polarity varies from cell type to cell type (Buckley and Johnston, 2022). A difference in the cargo sorting mechanisms or even the cargo themselves expressed by each of the polarized cells may also give rise to this variance between cell types. Based on the differences in proteomic and miRNA content, polarized exosomes can have a multitude of functions, depending on the cell type and the surface from which exosomes are released. Only a few research studies have characterized the function of contents of exosomes derived from polarized cells.

In RPE cells, the apical exosome was found to specifically contain αB crystallin (Sreekumar et al., 2010; Klingeborn et al., 2017), a neuroprotective protein. Administration of apical exosomes to RPE cells alleviated apoptosis caused by oxidative stress, possibly owing to internalization of αB crystallin in exosomes (Sreekumar et al., 2010). Other functions relatable to other cargo in apical exosomes or any function of basolateral exosomes have not been examined.

PTEC releases basolateral exosomes upon interferon treatment, which mimics inflammation. These exosomes are then taken up by monocytes, which induces chemokine secretion by the receptor monocytes (Wang et al., 2021). Although the molecules responsible for this function have not been identified, proteomic and miRNA analysis have narrowed down the candidates to a few proteins and miRNAs, including upregulated C-C motif chemokine ligand 5 (CCL5), intercellular adhesion molecule 1 (ICAM1), bone marrow stromal cell antigen 2 (BST2), and downregulated miR-30c-5p. Apical exosomes released upon inflammation had higher levels of inflammation-related miRNAs and proteins that might be related to transmit inflammatory signals to downstream tubules. Further evidence on uptake of apical exosomes and downstream cellular responses is awaited.

Exosomes released from two sides of polarized cholangiocytes were found to have different targets and different signaling characteristics (Davies et al., 2020). Apical and basolateral exosomes were administered to putative target cells or cell lines predicted from anatomical proximity before analysis of signal transduction. Apical exosomes from cholangiocytes seemed to target cells of the same type, as various signaling cascades were activated specifically in response to exosome administration. Among the cascades upregulated by apical exosomes were stress-activated protein kinase (SAPK) and extracellular signal-regulated kinase (ERK), pathways important in stress response and differentiation. Basolateral exosomes targeted a different type of cell, monocyte cell lines that were meant to model liver-resident macrophages. In these cells, basolateral exosomes upregulated the mammalian target of rapamycin (mTOR) and p53 cascades responsible for cell growth and proliferation. Exosome administration *vice versa* resulted in less or no changes in these pathways in both cell types, indicating the specificity of these exosomes and their receptor cells (Davies et al., 2020).

Investigation of the functional differences of apical and basolateral exosomes is a relatively new topic, but these reports have already revealed interesting differences in composition and function of different types of exosomes produced by polarized cells. A wider study is crucial for a further understanding of how each subtype of exosome affects other cells.

## 6 Perspectives: possible applications and challenges

As the regulation and functions of different subsets of exosomes in nonpolarized cells remain unclear, insights in those from different sides of polarized cells may give us hints. In other words, more research in polarized cells should unveil potentially druggable targets and possible uses of different exosome subtypes, although there are challenges one must overcome to clarify these details.

Research on functions of exosomes is not merely a matter of curiosity but a world full of potentially druggable targets, as more and more evidence relating specific exosomes to pathogenesis or disease progression are emerging. Polarized cells are not an exception, as neuronal (Huang et al., 2022) and epithelial (Pan et al., 2023) exosomes are being implicated in various diseases. Furthermore, Rab39B, one of the two isoforms of the Rab39 protein regulating basolateral exosome trafficking (Matsui et al., 2022), is a causative gene product of Parkinson’s disease (Wilson et al., 2014; Lesage et al., 2015; Mata et al., 2015), further fortifying the links found between Parkinson’s disease and exosomes (Danzer et al., 2012; Toro et al., 2015; Guo et al., 2020; Pinnell et al., 2021; Kluge et al., 2022; Li et al., 2022), and suggesting another link to those from polarized cells. A deeper investigation on how exosomes are involved in disease onset or progression should surely be beneficial for future intervention and prevention of these diseases.

As the proteins involved in apical or basolateral exosome trafficking and release are different (Matsui et al., 2022; 2023), these proteins may serve as novel druggable candidates once the involvement of apical or basolateral exosomes are revealed. As the pool of pole-specific proteins grow, the possibility of being able to target them increases. Although there are currently no drugs in clinical use against Rab proteins, Rab GTPases were found to be druggable as an inhibitor of Rab27a has been identified to inhibit neutrophil exocytosis (Johnson et al., 2016). Inhibitors against Ras proteins, a family of small GTPases relatively close to Rab proteins, have also started to emerge for cancer treatment (Yin et al., 2023). It could be a matter of time before any apically or basolaterally specific exosome inhibitor is developed.

Before all these ideal goals are achieved, there are multiple difficulties that need to be resolved. One major setback in all the cell types is the problem of quantity in obtaining exosomes from different poles. Physical separation of apical and basolateral exosomes involves a tight sheet of cells on cell culture inserts, making it difficult for scaling the system. As virtually no marker protein is known to be capable of isolating exosome subsets that originate from one side of the cell [or even exosomes themselves (Mathieu et al., 2021; Nikoloff et al., 2021)], some technical advances will be needed. Related to this, polarized cells without tight junctions (such as neurons or some types of activated immune cells) could not be assessed by the same approach. Other challenges involve cellular functions that depend on receptor cells. Although it might be expected that cells anatomically nearby are the receptor cells, some subsets of exosomes are known to circulate and target specific tissues far away (Hoshino et al., 2015).

There are still gaps between the typical schema of exosome analysis, i.e., proteomic or miRNA analysis of exosomes from body fluids, which is basically exosomes from unknown origin, and the analysis of exosome biogenesis, i.e., observing changes in the quantity or content of exosomes of unknown function. A seamless cooperation between clinicians and researchers should surely fill in the gap, and polarized cells have the potential to be a keystone in this process. Somewhere ages and ages hence, we might be able to tell the story that a breakthrough was made by the “two roads” diverged in a cell.
